# Potential Therapeutic Mechanism of Traditional Chinese Medicine on Diabetes in Rodents: A Review from an NMR-Based Metabolomics Perspective

**DOI:** 10.3390/molecules27165109

**Published:** 2022-08-11

**Authors:** Yinli Huang, Jiahui Lu, Qihui Zhao, Junli Chen, Wei Dong, Minjie Lin, Hong Zheng

**Affiliations:** 1 Department of Endocrinology, Pingyang Affiliated Hospital of Wenzhou Medical University, Wenzhou 325400, China; 2 School of Pharmaceutical Sciences, Wenzhou Medical University, Wenzhou 325035, China

**Keywords:** amino acid, antidiabetic, metabolomics, energy metabolism, ketone body

## Abstract

Traditional Chinese medicine (TCM) has been used to treat diabetes for a long time, but its application has not been widely accepted due to unstandardized product quality and complex pharmacological mechanisms. The modernization of TCM is crucial for its further development, and in recent years the metabolomics technique has largely driven its modernization. This review focuses on the application of NMR-based metabolomics in diabetic therapy using TCM. We identified a series of metabolic pathways that altered significantly after TCM treatment, providing a better understanding of the metabolic mechanisms of TCM for diabetes care.

## 1. Introduction

Diabetes is a common metabolic disease characterized by hyperglycemia owing to insulin secretion deficiency for type 1 diabetes (T1D) or insulin resistance for type 2 diabetes (T2D), which has become a global health problem [1]. In 2021, approximately 537 million adults between 20 and 79 years of age suffered from diabetes worldwide, and this number is projected to increase to 783 million by 2045 [1]. More than three out of every four diabetic patients were living in low- and middle-income countries. Moreover, diabetes caused 6.7 million deaths in 2021 [1]. Currently, T2D can be treated by a number of different medications such as metformin, sulfonylureas, glinides, thiazolidinediones, DPP-4 inhibitors, GLP-1 receptor agonists and SGLT2 inhibitors. However, there are fewer treatment methods for T1D, so all T1D patients require daily insulin injections to maintain normal blood glucose levels. Therefore, there is an urgent need to discover novel therapeutic strategies, especially for T1D. Traditional Chinese medicine (TCM) is a system of healing that originated thousands of years ago that has also been used to treat diabetes for a long time [2,3,4]. However, several problems including unstandardized product quality and complex pharmacological mechanisms have restricted its wide acceptance and application [5]. Therefore, TCM modernization is crucial for its further development [6]. This review aims to provide the currently available information on potential metabolic mechanisms of TCM on the management and treatment of diabetes for diabetic patients, pharmacologists, drug developers and endocrinologists.

## 2. Metabolomics as a Powerful Tool for the Modernization of TCM

**Figure 1:**
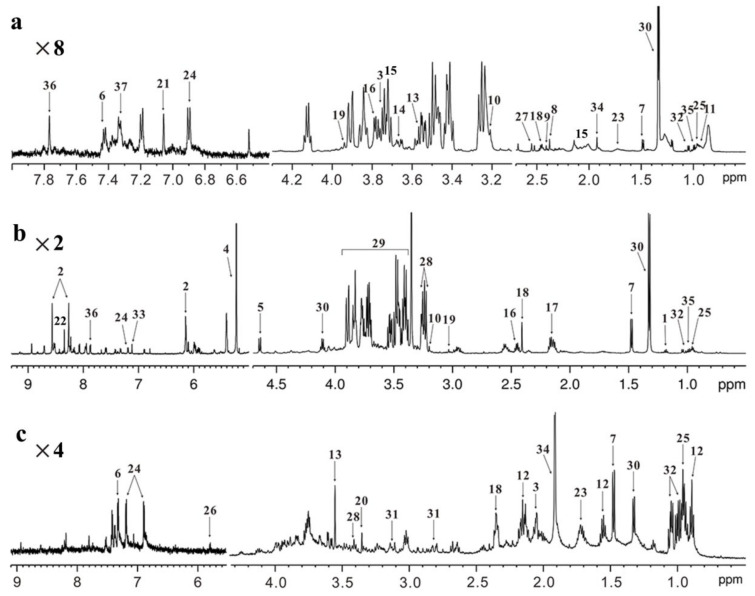
NMR-based metabolomics profiling. Typical 600 MHz ^1^H NMR spectra obtained from (**a**) serum, (**b**) liver and (**c**) feces in healthy mice. Metabolite assignment: 1, 3-hydroxybutyrate; 2, AMP; 3, NAG; 4, α-glucose; 5, β-glucose; 6, phenylalanine; 7, alanine; 8, acetone; 9, pyruvate; 10, choline; 11, LDL/VLDL; 12, butyrate; 13, glycine; 14, glycerol; 15, glutamate; 16, glutamine; 17, glutathione; 18, succinate; 19, creatine; 20, methanol; 21, methylhistidine; 22, formate; 23, lysine; 24, tyrosine; 25, leucine; 26, uracil; 27, citrate; 28, taurine; 29, glucose/amino acid region; 30, lactate; 31, aspartate; 32, valine; 33, fumarate; 34, acetate; 35, isoleucine; 36, histidine; 37, tryptophan. Amplification: ×2, 2 times; ×4, 4 times; ×8, 8 times.

In recent years, omics technologies have largely driven the modernization of TCM [7]. Metabolomics is the apogee of the omics cascade that attempts to analyze a comprehensive set of metabolites in biological samples and explore changes in metabolic pathways related to genomic and proteomic perturbations [8]. TCM possesses several typical characteristics such as being multi-component, multi-target and multi-pathway, resulting in great difficulty when attempting to explore its pharmacological mechanisms [9]. Notably, metabolomics, especially untargeted metabolomics, can detect a global set of metabolites without bias in living organisms after TCM treatment, which provides the possibility of exploring the metabolic mechanisms of TCM in disease prevention and treatment [10]. Currently, two analytical platforms are mainly employed to acquire metabolomic data, including mass spectrometry (MS) and nuclear magnetic resonance (NMR) spectroscopy [11]. These techniques have their advantages and disadvantages, as listed in Table 1. For example, the MS-based method has a higher sensitivity and more metabolites can be detected even using a minimal sample size. Moreover, the MS-based method is also a flexible technique, which can couple liquid or gas chromatography to achieve the selection and separation of different metabolites. However, it also has a number of disadvantages including low reproducibility, complex sample preparation, non-recyclable samples, relatively poor quantitative analysis and difficult metabolite identification. The NMR-based method possesses several strengths, such as high reproducibility, simple sample preparation, non-destructive, fast analysis, good quantitative analysis and straightforward identification, although this method cannot analyze non-protonated metabolites. In addition, compared with the MS-based method, NMR analysis needs a larger sample size and has a relatively low sensitivity. Figure 1 shows the typical ^1^H NMR-based metabolomics profiling obtained from serum, liver and feces samples in healthy mice [12], and the detailed metabolite assignments are listed in Table 2, where a series of metabolites can be identified involving amino acid metabolism, energy metabolism, fatty acid metabolism, ketone body metabolism and others. NMR metabolomics profiling is tissue-specific due to different metabolite compositions. Therefore, different analytical sequences have been developed for NMR analysis. For example, a Carr–Purcell–Meiboom–Gill (CPMG) sequence is usually conducted for serum samples in order to minimize the line-broadening effect of blood macromolecules including proteins and lipids. However, for samples with a high water content such as urine, a standard single-pulse sequence (ZGPR) can be used to reduce the impact of water signals on metabolomics profiling. In addition, there is a greater likelihood of overlapping peaks from multiple metabolites with NMR analysis, resulting in difficult identification and quantification. One way to solve this problem is to perform NMR experiments under higher magnetic fields. Moreover, 2D J-resolved spectroscopy and spectral deconvolution have also been used to address the peak overlap of metabolites.

**Figure 2:**
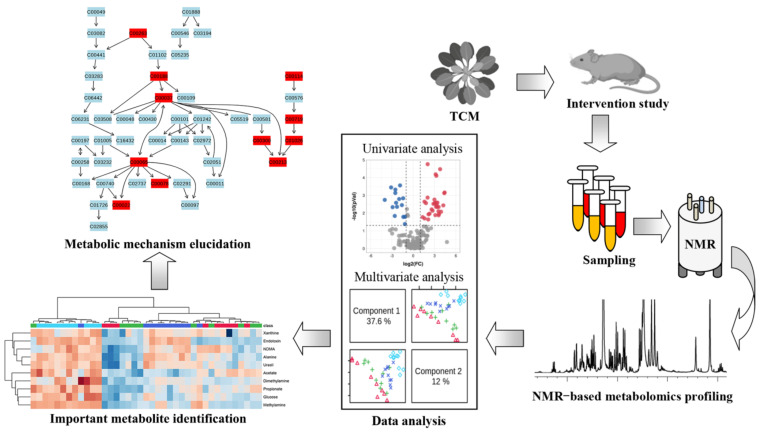
Flowchart depicting NMR-based metabolomics method to elucidate metabolic mechanisms of traditional Chinese medicine for the treatment of diseases.

In this review, we focus on the application of NMR metabolomics in diabetes therapy using TCM, providing a better understanding of the metabolic mechanisms of TCM. Figure 2 illustrates the flowchart of the NMR-based metabolomics method for elucidating the metabolic mechanisms of TCM for diabetes treatment. In brief, diabetic rodent models are treated with TCM after a period of time and then biological samples are collected for NMR-based metabolomics analysis, such as serum, plasma, urine, feces and tissue samples. Metabolomics data are subjected to multivariate and univariate analyses to identify important metabolites that are significantly altered after TCM treatment. Finally, metabolic pathway analysis is performed to elucidate potential therapeutic mechanisms of TCM for diabetes.

## 3. Potential Metabolic Mechanisms of TCM on Diabetes Care

**Figure 3:**
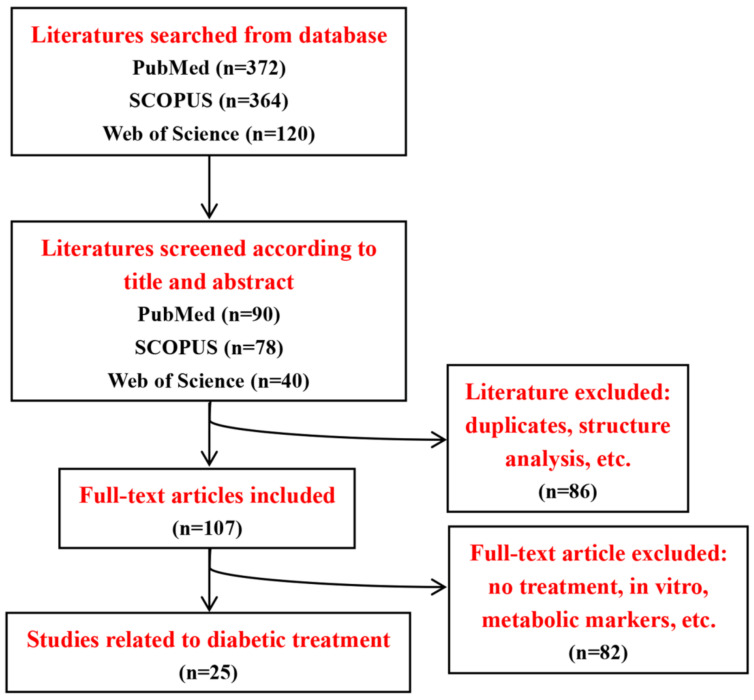
Flowchart of literature search and selection.

A systematic search of the PubMed, SCOPUS and Web of Science databases was conducted for relevant studies from 2012 to 2022. Different possible combinations of the following search terms were used: “traditional Chinese medicine”, “Chinese medicine”, “TCM”, “diabetes”, “diabetic”, “nuclear magnetic spectroscopy”, “NMR”, “metabolomic”, “metabonomic” and “metabolic”. We independently searched the literature to minimize bias and firstly screened studies according to titles and abstracts. Inclusion and exclusion criteria were discussed and defined for literature selection. The following inclusion criteria were used: Firstly, studies should be related to diabetes, NMR-based metabolomic analysis, TCM treatment and rodents. Secondly, studies need to measure a specific metabolite level and compare the metabolic differences in diabetes with and without TCM treatment. The exclusion criteria included drug structure analysis, in vitro studies, biomarker discovery or pathological studies. Moreover, reviews, meta-analyses, abstracts and case reports were also excluded. The detailed procedure of literature selection is illustrated in Figure 3, and the information of 25references included in this review are listed in Table 3, of which there are 17 references on T2D and 8 references on T1D. In animal studies, T1D models were developed by streptozotocin or alloxan induction, but lacked non-obese diabetic (NOD) mouse models. Several T2D animal models were used including high-fat diet-fed and low-dose streptozotocin-treated models, KKay mice and Zucker diabetic rats, whereas the use of db/db mice as a widely used preclinical model of T2D also needs to be considered for future studies.

**Figure 4:**
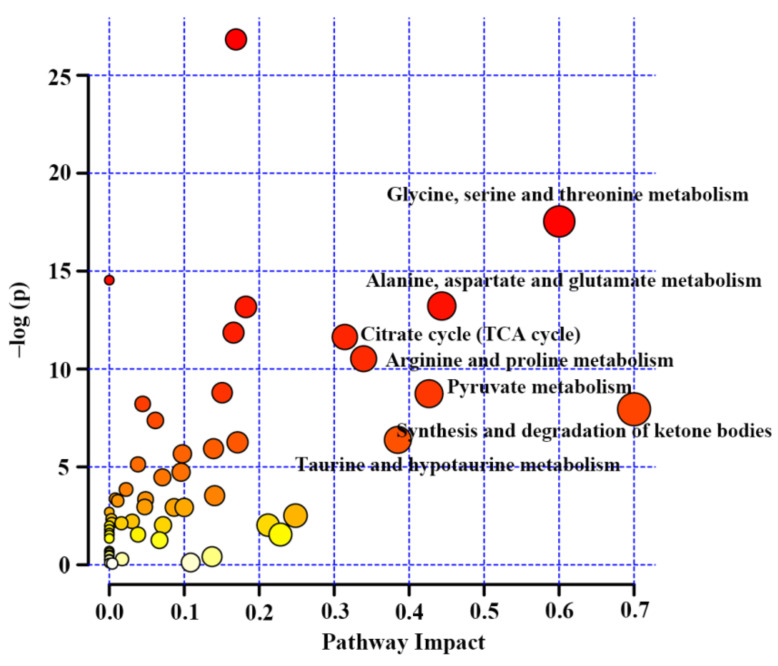
Metabolic pathway analysis based on differentiated metabolites from NMR metabolomics studies on diabetic treatment using traditional Chinese medicine.

Subsequently, metabolic pathway analysis was carried out on the basis of the metabolites included in this review by the MetaboAnalyst 5.0 [38]. The result of pathway analysis was presented according to −log (*p*) values from the pathway enrichment analysis and pathway impact values from the pathway topology analysis. A metabolic pathway with high values of these two parameters was identified as the important pathway in the response to TCM treatment, as shown in Figure 4.

### 3.1. Amino Acid Metabolism

**Figure 5:**
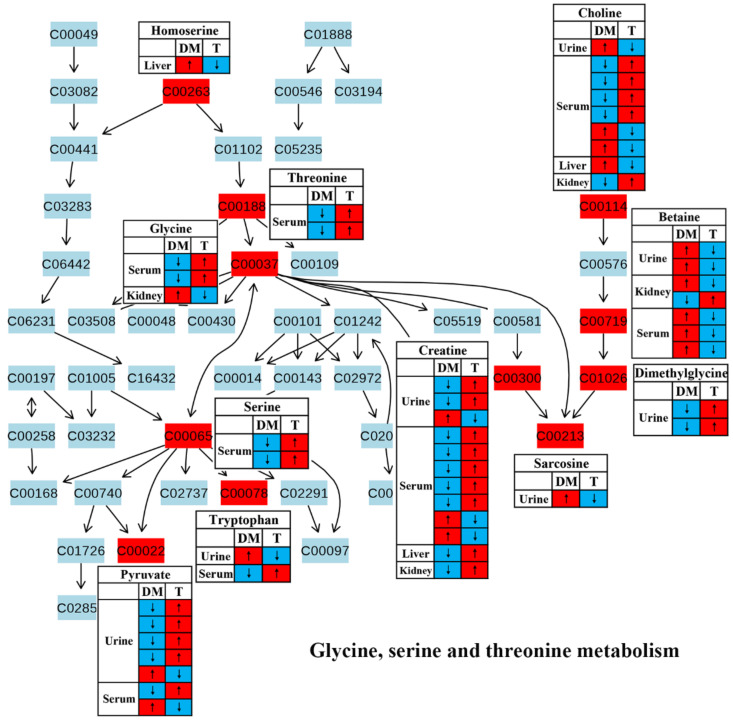
The effect of traditional Chinese medicine onglycine, serine and threonine metabolism during diabetic treatment. Each row in the table represents one study and arrow indicates relative change tendency of metabolite. Red and wathet blue colors indicate the increase and decrease in metabolite level in DM relative to normal controls or in DM after TCM treatment, respectively. DM, diabetes mellitus; T, TCM treatment. Metabolite: C00022, pyruvate; C00037, glycine; C00065, serine; C00078, tryptophan; C00114, choline; C00188, threonine; C00213, sarcosine; C00263, homoserine; C00300, creatine; C00719, betaine; C01026, dimethylglycine.

**Figure 6:**
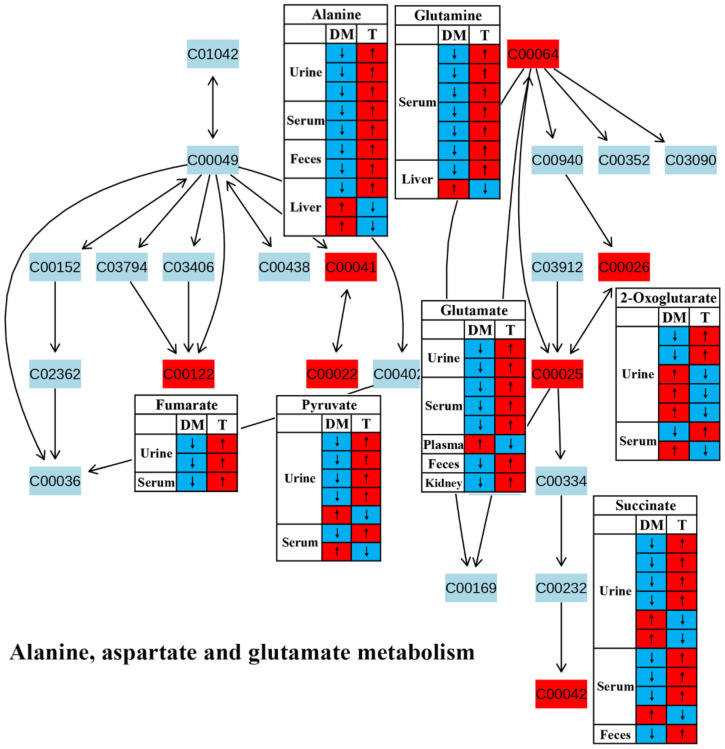
The effect of traditional Chinese medicine on alanine, aspartate and glutamate metabolism during diabetic treatment. Each row in the table represents one study and arrow indicates relative change tendency of metabolite. Red and wathet blue colors indicate the increase and decrease in metabolite level in DM relative to normal controls or in DM after TCM treatment, respectively. DM, diabetes mellitus; T, TCM treatment. Metabolite: C00022, pyruvate; C00025, glutamate; C00026, 2-oxoglutarate; C00041, alanine; C00042, succinate; C00064, glutamine; C00122, fumarate.

**Figure 7:**
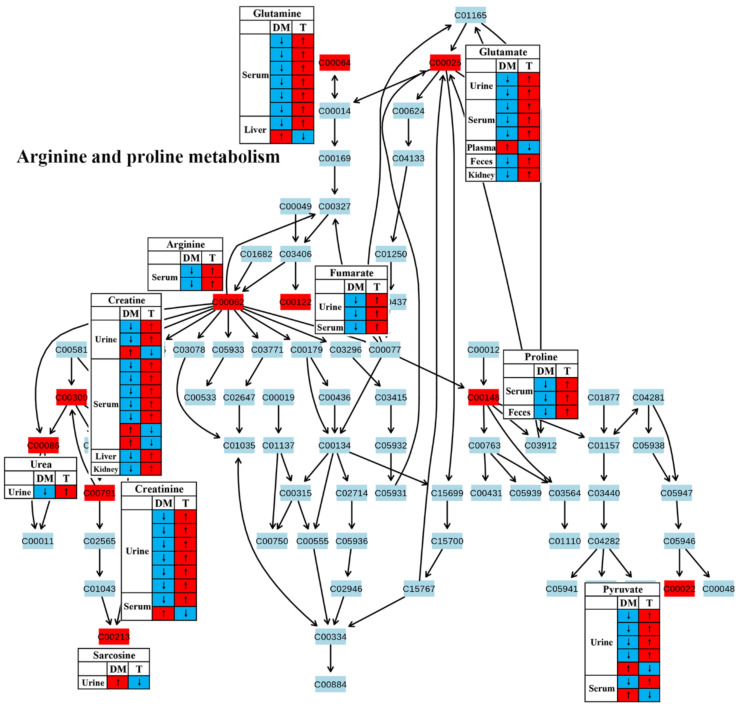
The effect of traditional Chinese medicine on arginine and proline metabolism during diabetic treatment. Each row in the table represents one study and arrow indicates relative change tendency of metabolite. Red and wathet blue colors indicate the increase and decrease in metabolite level in DM relative to normal controls or in DM after TCM treatment, respectively. DM, diabetes mellitus; T, TCM treatment. Metabolite: C00022, pyruvate; C00025, glutamate; C00062, arginine; C00064, glutamine; C00086, urea; C00122, fumarate; C00148, proline; C00213, sarcosine; C00300, creatine; C00791, creatinine.

Amino acid metabolism has been reported to play a key role in insulin secretion and thereby affect the onset and development of diabetes [39]. In this review, most studies reported a reduced amino acid metabolism in diabetic rodents, including glycine, serine and threonine metabolism (Figure 5), alanine, aspartate and glutamate metabolism (Figure 6) and arginine and proline metabolism (Figure 7). We found that the changes in these amino acids might be associated with the regulation of insulin signaling. For example, Wang-Sattler et al. revealed that a lower glycine level could be a predictor for impaired glucose tolerance and T2D [40]. Glycine can increase insulin sensitivity by suppressing oxidative stress in sucrose-fed rats [41]. In addition, serine/threonine phosphorylation plays an essential role in the regulation of pancreatic β-cell growth/survival and insulin signaling [42,43]. Brennan et al. revealed that alanine increased insulin secretion via the physiological regulation of β-cell electrical activity [44]. Alanine can oxidize to glutamate in β-cells [44], and glutamate serves as an intracellular messenger in the regulation of insulin secretion in response to glucose [45]. In a supplementation trial, glutamate has also been evidenced to improve glucose metabolism by increasing insulin secretion in healthy males [46]. Moreover, Gheni et al. elucidated that glutamate derived from the malate-aspartate shuttle is a key signal between glucose metabolism and cAMP action in incretin-induced insulin secretion [47]. Monti et al. conducted a human intervention study for 18 months and found that arginine supplementation significantly increased regression to normal glucose tolerance, although there was no significant effect on the incidence of diabetes [48]. In addition, arginine has also been reported to perform an essential role in pancreatic β-cell functional integrity [49] and improve insulin sensitivity [50]. In this review, we found that the metabolism of these aminoacids was up-regulated after TCM treatment in most studies, suggesting that TCM may improve insulin action and glycemic control via the regulation of amino acid metabolism.

### 3.2. Energy Metabolism

**Figure 8:**
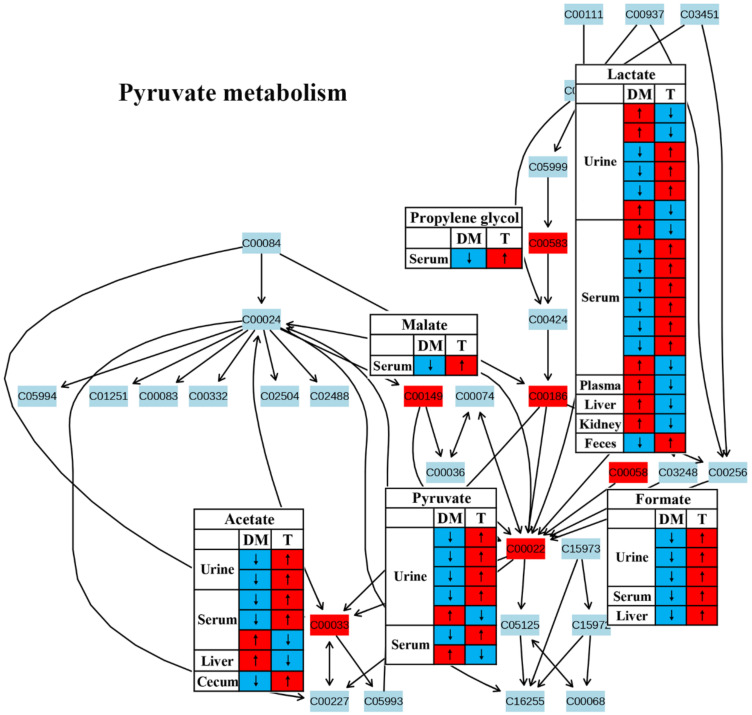
The effect of traditional Chinese medicine on pyruvate metabolism during diabetic treatment. Each row in the table represents one study and arrow indicates relative change tendency of metabolite. Red and wathet blue colors indicate the increase and decrease in metabolite level in DM relative to normal controls or in DM after TCM treatment, respectively. DM, diabetes mellitus; T, TCM treatment. Metabolite: C00022, pyruvate; C00033, acetate; C00058, formate; C00149, malate; C00186, lactate; C00583, propylene glycol.

**Figure 9:**
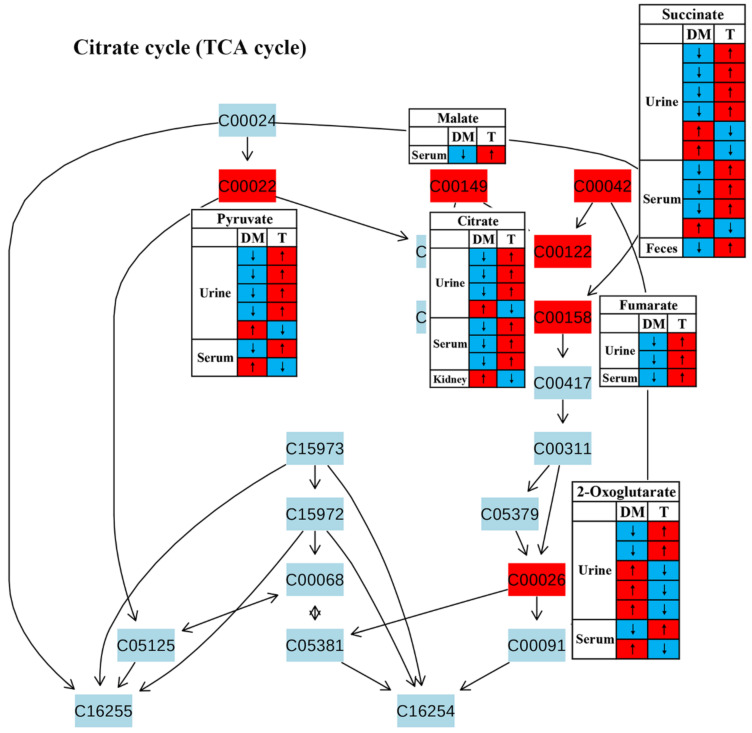
The effect of traditional Chinese medicine on TCA cycle during diabetic treatment. Each row in the table represents one study and arrow indicates relative change tendency of metabolite. Red and wathet blue colors indicate the increase and decrease in metabolite level in DM relative to normal controls or in DM after TCM treatment, respectively. DM, diabetes mellitus; T, TCM treatment. Metabolite: C00022, pyruvate; C00149, malate; C00042, succinate; C00122, fumarate; C00158, citrate; C00026, 2-oxoglutarate.

In this review, decreases in pyruvate metabolism (Figure 8) and the TCA cycle (Figure 9) in diabetic rodents were reported by most studies, which confirm that mitochondrial dysfunction occurs in diabetes. Mitochondrial damage has been associated with pancreatic β-cell dysfunction and insulin resistance, resulting in the abnormal glucose metabolism of diabetes [51,52,53,54]. However, notably, treatment with TCM can up-regulate these two metabolic pathways in diabetic rodents, suggesting that TCM may alleviate the mitochondrial dysfunction induced by diabetes. Moreover, glucose as a main source for energy production can also be converted to pyruvate, and then pyruvate oxidized to CO_2_ and H_2_O via the tricarboxylic acid cycle (TCA cycle). Therefore, increased energy metabolism boosts glucose depletion, which might be a possible mechanism of the glucose-lowering effect of TCM treatment.

### 3.3. Synthesis and Degradation of Ketone Bodies

**Figure 10:**
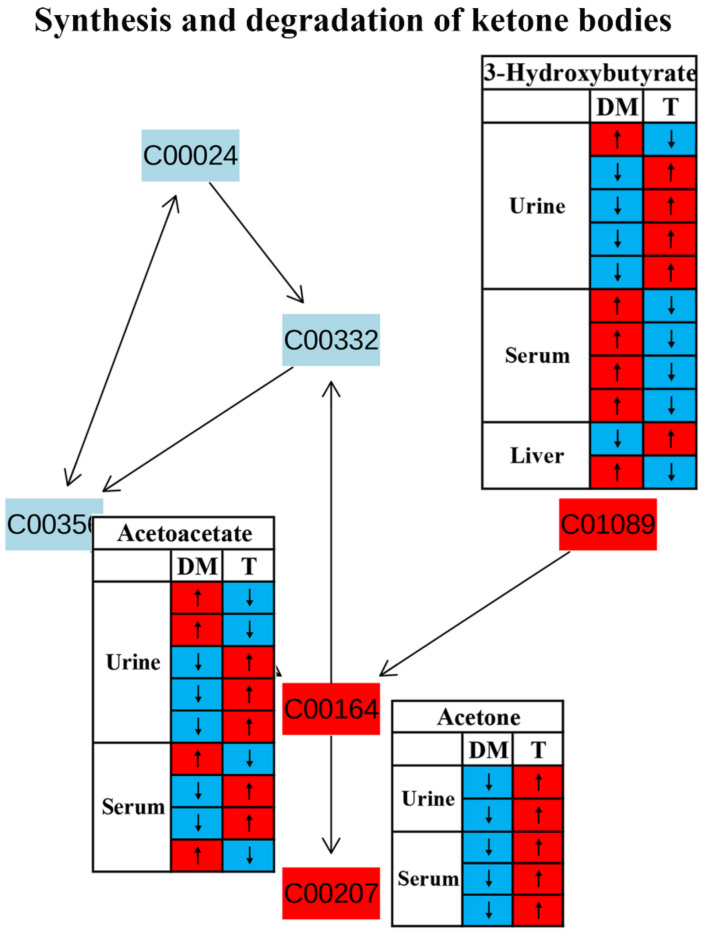
The effect of traditional Chinese medicine on synthesis and degradation of ketone bodies during diabetic treatment. Each row in the table represents one study and arrow indicates relative change tendency of metabolite. Red and wathet blue colors indicate the increase and decrease in metabolite level in DM relative to normal controls or in DM after TCM treatment, respectively. DM, diabetes mellitus; T, TCM treatment. Metabolite: C00207, acetone; C00164, acetoacetate; C01089, 3-hydroxybutyrate.

We also identified significant changes in the synthesis and degradation of ketone bodies in response to TCM treatment (Figure 4). Ketone bodies are derived from fatty acid metabolism and mostly generated in the liver as an alternative source of energy [55]. Their homeostasis is maintained by the balance of synthesis (ketogenesis) and degradation (ketolysis) of ketone bodies. Ketogenesis is the process of converting fatty acids into two major ketonebodies, acetoacetate and β-hydroxybutyrate [56]. However, changes in acetoacetate and β-hydroxybutyrate in diabetic rodents after TCM treatment were inconsistent based on the current findings using NMR metabolomics (Figure 10). The levels of ketone bodies can also be regulated by insulin; for example, an elevated insulin level promotes ketone body clearance by increasing their catabolic pathway in extrahepatic tissues [57]. Thus, enhanced ketone body production was observed in diabetes patients owing to insulin insufficiency or resistance [58]. Notably, the level of acetone in the urine and serum was significantly reduced in diabetic rodents but increased after TCM treatment in all studies included in this review (Figure 10), suggesting an enhanced ketone body degradation since acetone is produced via the decarboxylation of acetoacetate [56]. Nevertheless, the causal relationship between ketone body degradation and insulin signaling after TCM treatment still needs further confirmation.

### 3.4. Taurine and Hypotaurine Metabolism

**Figure 11:**
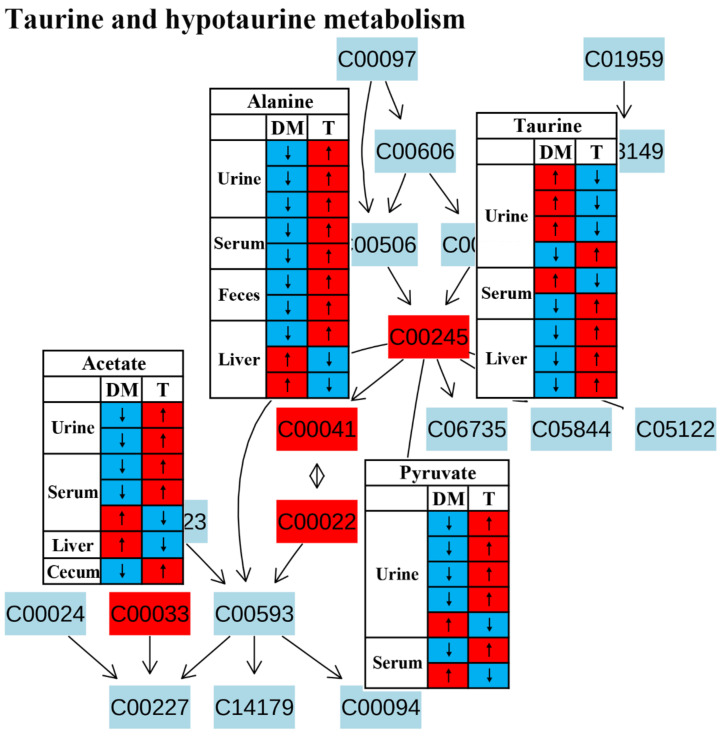
The effect of traditional Chinese medicine on taurine and hypotaurine metabolism during diabetic treatment. Each row in the table represents one study and arrow indicates relative change tendency of metabolite. Red and wathet blue colors indicate the increase and decrease in metabolite level in DM relative to normal controls or in DM after TCM treatment, respectively. DM, diabetes mellitus; T, TCM treatment. Metabolite: C00022, pyruvate; C00033, acetate; C00041, alanine; C00245, taurine.

Metabolic pathway analysis suggested that taurine and hypotaurine metabolism was affected after TCM treatment (Figure 4).Taurine has been reported to restore insulin secretion and exert an antidiabetic effect [59,60,61].In this review, the level of taurine in the urine was increased in diabetic rodents but reduced after TCM treatment in most studies, as shown in Figure 11.However, the level of taurine was decreased in the livers of diabetic rodents and increased by the administration of TCM such as *Dendrobium officinale* water extract [20] and the Qijian mixture [26], which could be beneficial to improve insulin signaling in the liver [62]. Carneiro et al. revealed that taurine facilitated glucose homeostasis by regulating the expression of genes for glucose-stimulated insulin secretion [63]. Additionally, taurine can also affect the electrogenic response and calcium homeostasis in β-cells and then result in insulin secretion [64]. Although the current findings on taurine metabolism are inconsistent, we speculate that an increased taurine level after TCM treatment should have a positive effect on diabetes therapy.

## 4. Conclusions and Perspectives

**Figure 12:**
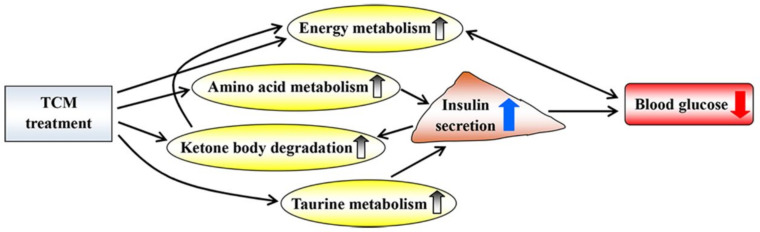
Potential metabolic mechanisms of traditional Chinese medicine on diabetic treatment. Up and down arrows indicate increase and decrease after TCM treatment, respectively.

This review has focused mostly on NMR metabolomics and provides a panoramic view of the metabolic responses to diabetic treatment using TCM. Treatment with TCM up-regulates energy metabolism, amino acid metabolism, ketone body degradation and taurine metabolism and then increases insulin secretion and reduces blood glucose levels (Figure 12). However, the relevant studies are still inadequate to make a final conclusion. Moreover, the real knowledge for a complex biological system needs to integrate genes, proteins and metabolites, suggesting that a multi-omics analysis could be one of the avenues to explore in the future. In this review, most studies focused on analyses of biofluids, such as urine and serum, but we suggest that attention should also be paid to metabolic changes in organs and tissues in order to explore potential mechanisms underlying the treatment of diabetic complications using TCM. Notably, the fecal metabolome also needs to be paid more attention in order to explore the role of gut microbiota in diabetes therapy via TCM treatment [65].

Thus far, the clinical studies of TCM in diabetes have mostly focused on glycemic control, but with no metabolomics investigations. Thus, metabolic pathways affected by TCM treatment in rodents still need to be validated in human intervention studies in order to uncover potential pharmacological mechanisms of TCM for its clinical translational application. We also recommend using a multi-omics analysis for elucidating whether these metabolic changes have a causative role in diabetes therapy. Such metabolomics information could then be used to evaluate TCM interventions and discover new targets for diabetic treatment. Relative to T2D, there are fewer therapeutic methods for T1D, but there are few TCM studies on T1D. We appeal for the need to discover novel therapeutic strategies for T1D patients from TCM in the future.

## Figures and Tables

**Table 1:** Summary of the main advantages and disadvantages of nuclear magnetic resonance (NMR) spectroscopy and mass spectrometry (MS) in metabolomics.

	NMR	MS
Advantage	High reproducibility	High sensitivity
	Minimal sample preparation	More metabolite detection
	Non-destructive	Flexible technique
	Good quantitative analysis	Minimal sample size
	No separation and fast analysis	
	Good software/database for identification	
Disadvantage	Relatively low sensitivity	Low reproducibility
	Larger sample size	Sample derivatization for GC-MS
	Cannot detect non-protonated metabolites	Sample not recoverable
		Relatively poor quantitative analysis
		Difficult identification

**Table 2:** Metabolite assignment in ^1^H NMR-based metabolomics profiling.

No.	Metabolite	Chemical Shift (ppm) ^a^	Metabolic Pathway
1	3-Hydroxybutyrate	1.18(d)	Ketone body metabolism
2	AMP ^b^	6.15(d), 8.26(s), 8.58(s)	Energy metabolism
3	NAG ^c^	2.05(m), 3.75(m)	- ^e^
4	α-Glucose	5.21(d)	Energy metabolism
5	β-Glucose	4.65(d)	Energy metabolism
6	Phenylalanine	7.37(t), 7.45(t)	Amino acid metabolism
7	Alanine	1.48(d)	Amino acid metabolism
8	Acetone	2.37(s)	Ketone body metabolism
9	Pyruvate	2.40(s)	Energy metabolism
10	Choline	3.20(s)	Choline metabolism
11	LDL/VLDL ^d^	0.85(m), 1.25(m)	-
12	Butyrate	0.89(t), 1.55(m)	Fatty acid metabolism
13	Glycine	3.55(s)	Amino acid metabolism
14	Glycerol	3.67(q)	Glycerolipid metabolism
15	Glutamate	2.15(m), 3.75(m)	Amino acid metabolism
16	Glutamine	2.45(m), 3.78(t)	Amino acid metabolism
17	Glutathione	2.15(m)	Amino acid metabolism
18	Succinate	2.39(s)	Energy metabolism
19	Creatine	3.03(s), 3.93(s)	Energy metabolism
20	Methanol	3.35(s)	-
21	Methylhistidine	7.05(s)	Amino acid metabolism
22	Formate	8.44(s)	Fatty acid metabolism
23	Lysine	1.71(m)	Amino acid metabolism
24	Tyrosine	6.89(d), 7.20(d)	Amino acid metabolism
25	Leucine	0.95(t)	Amino acid metabolism
26	Uracil	5.80(d)	Nucleotide metabolism
27	Citrate	2.55(d)	Energy metabolism
28	Taurine	3.25(t), 3.41(t)	Amino acid metabolism
29	Glucose/amino acid region	3.35–3.92(m)	-
30	Lactate	1.32(d), 4.11(q)	Energy metabolism
31	Aspartate	2.80(d), 3.15(d)	Amino acid metabolism
32	Valine	0.98(d), 1.05(d)	Amino acid metabolism
33	Fumarate	7.11(s)	Energy metabolism
34	Acetate	1.91(s)	Fatty acid metabolism
35	Isoleucine	0.99(d)	Amino acid metabolism
36	Histidine	7.79(s)	Amino acid metabolism
37	Tryptophan	7.34(d)	Amino acid metabolism

^a^ s, singlet; d, doublet; t, triplet; q, quartet; m, multiplet; ^b^ adenosine monophosphate; ^c^ *n*-acetyl-glycoprotein; ^d^ low-density lipoprotein/very low-density lipoprotein; ^e^ others.

**Table 3:** Summary of main metabolic changes after TCM treatment.

Treatment	Dose/Time	Model	Type	Glucose Lowering	Sample	Metabolic Change ^a^	Reference
Zhibai Dihuang pill	4 g/kg; 30 days	STZ-induced diabetic nephropathy rats	T1D	Yes, but no significant difference	Urine Serum Kidney	Urine: (↓)3-hydroxybutyrate, lactate Serum: (↑) creatine, methionine, lactate, pyruvate; (↓) VLDL/LDL, 3-hydroxybutyrate Kidney: (↑) betaine, choline, glutamate; (↓)glucose, lactate	[13]
Gegen Qinlian decoction	8 g/kg; 5 weeks	High-fat diet/STZ-induced diabetic rats	T2D	Yes	Plasma	(↑)lipoprotein, valine, TMAO, dimethylamine, arginine; (↓) choline, glucose, glycerol, taurine, creatine, creatinine, tyrosine	[14]
*Momordica charantia* ethanol extract	200 mg/kg; 1 week	STZ-induced diabetic rats	T1D	Yes	Urine	(↑) succinate, creatine, creatinine, urea, phenylacetylglycine; (↓) lactate, glucose	[15]
*Phyllanthus niruri* ethanol extract	500 mg/kg; 4 weeks	High-fat diet/STZ-induced diabetic rats	T2D	Yes	Urine Serum	Urine: (↑) hippurate, formate, fumarate, methylnicotinamide, pyruvate, acetone, phenylacetylglycine, allantoin, alanine, succinate, lactate; (↓) glucose, choline, taurine, creatine Serum: (↓) glucose, triglyceride, cholesterol, LDL, HDL	[16]
*Andrographis paniculata* water extract	200 mg/kg; 4 weeks	High-fat diet/STZ-induced diabetic rats	T2D	Yes	Urine	(↑) lactate, formate, pyruvate, citrate, 2-oxoglutarate, succinate, acetoacetate, 3-hydroxybutyrate, acetate, dimethylglycine, dimethylamine, alanine, allantoin; (↓) glucose, taurine	[17]
*Centella asiatica* ethanol extract	300 mg/kg; 4 weeks	High-fat diet/STZ-induced diabetic rats	T2D	Yes	Urine Serum	Urine: (↑) pyruvate, lactate, citrate, fumarate, succinate, 2-oxoglutarate, 3-hydoxybutyrate, acetoacetate, acetone, acetate, alanine, hippurate, dimethylamine, creatinine, trimethylamine, allantoin; (↓) glucose Serum: (↑) lactate, choline, succinate; (↓) glucose	[18]
Genipin, derived from the fruit of *Gardenia* *jasminoides*	100 mg/kg; 2 weeks	Alloxan-induced diabetic rats	T1D	Yes	Serum	(↑) citrate, succinate, 3-hydroxybutyrate, acetone	[19]
*Orthosiphon stamineus* aqueous extract	500 mg/kg; 2 weeks	STZ-induced diabetic rats	T1D	Yes	Urine	(↑) hippurate, allantoin, creatinine, glutamate, 3-hydroxybutyrate, pyruvate, citrate; (↓) glucose, taurine, betaine, leucine, acetoacetate	[20]
*Dendrobium officinale* water extract	700 mg/kg; 2 weeks	STZ-induced diabetic mice	T1D	Yes	Serum Liver	Serum: (↑) citrate, glutamine; (↓) glucose, creatine Liver: (↑) creatine, alanine, leucine, isoleucine, valine, glutamine, glutathione, taurine, 3-hydroxybutyrate	[21]
*Melicopelunu-ankenda* leaf ethanol extract	400 mg/kg; 8 weeks	High-fat diet/STZ-induced diabetic rats	T2D	Yes	Serum	(↑) lactate, formate, 2-oxoglutarate, succinate, leucine, isoleucine, hippurate; (↓) glucose, acetoacetate, 3-hydroxybutyrate, choline, creatine	[22]
*Ipomoea aquatic* ethanolic extract	250 mg/kg; 4 weeks	High-fat diet/STZ-induced diabetic rats	T2D	Yes, but no significant difference	Urine	(↑)creatine, creatinine, hippurate, leucine, 1-methylnicotinamice, taurine, 3-hydroxybutyrate, lysine, trigonelline, allantoin, formate; (↓) glucose, citrate, carnitine, 2-oxoglutarate, succinate, tryptophan, acetoacetate, dimethylamine	[23]
Genipin, derived from the fruit of *Gardenia* *jasminoides*	100 mg/kg; 2 weeks	Alloxan-induced diabetic rats	T1D	Not mentioned	Urine Kidney	Urine: (↑) isoleucine, glutamate, acetoacetate, hippurate, N-acetyl-glycoprotein, creatinine, methylamine, dimethylglycine; (↓) 2-oxoglutarate, betaine, sarcosine Kidney: (↑) creatine; (↓) glycine, betaine	[24]
Zishen Jiangtang pill	3.0 g/kg; 8 weeks	STZ-induced rats with diabetic osteoporosis	T1D	Yes	Blood Urine	Blood: (↑) tryptophan, malate, propylene glycol, xanthosine, fumarate Urine: (↓) butyrate	[25]
Qijian mixture	5.385 g/kg; 8 weeks	Male KKay mice	T2D	Yes	Liver Kidney	Liver: (↑) glucose, taurine, glycerol; (↓) isoleucine, valine, lactate, alanine, acetate, homoserine, glutarate, 3-hydroxybutyrate, glutamine, glutathione, choline, anserine, niacinamide, xanthine, inosine Kidney: (↑) phosphocholine, TMAO, myo-inositol, xanthine; (↓) citrate	[26]
Mangiferin (SA1) and naringenin (SA2) from the leaves of *Salacia oblonga*	100 mg/kg; 15 days	STZ-induced diabetic rats	T2D	Yes	Serum	SA1: (↑) isoleucine, leucine, valine, lactate, alanine, acetate, proline, N-acetyl-glycoprotein, O-acetyl-glycoprotein, acetone, glutamate, glutamine, lipid, creatine, creatinine, malonate, choline, methanol, myo-inositol, serine, gluconate, threonine, allantoin, tyrosine, phenylalanine, histidine; (↓) glucose SA2: (↑) HDL/LDL, LDL/VLDL, isoleucine, leucine, valine, lactate, alanine, acetate, proline, N-acetyl-glycoprotein, O-acetyl-glycoprotein, acetone, glutamate, glutamine, lipid, creatine, malonate, choline, methanol, myo-inositol, glycerol, serine, gluconate, threonine, allantoin, tyrosine, phenylalanine, histidine; (↓) glucose	[27]
*Ganoderma lucidum* polysaccharides	400 mg/kg; 4 weeks	STZ-induced T2D rats	T2D	Yes	Feces	(↓) xanthine, deoxycholic acid, imidazole, *n*-Heptanoate, Urocanate, valine; (↑) methanol	[28]
*Rubus suavissimus* S. Lee	3 g/kg; 6 weeks	STZ-induced T1D rats	T1D	Yes	Urine	(↑) creatinine, allantoin, hippurate; (↓) lactate, pyruvate, succinate, 2-oxoglutarate, citrate	[29]
*Salvia miltiorrhiza* and *Radix Pueraria lobata* herb pair	3.15 g/kg; 4 weeks	STZ-induced T2D rats	T2D	Yes	Feces	(↑) alanine, succinate, lactate, proline, valine, leucine, glutamate, glucose, isoleucine, α-ketoisovalerate, hypoxanthine; (↓) butyrate	[30]
Anthocyanin Extracts from Bilberry and Purple Potato	25 and 50 mg/kg; 8 weeks	Zucker diabetic rats	T2D	Yes	Plasma	(↓) lactate, lipid, valine, leucine, isoleucine, glutamate	[31]
*Berberis kansuensis* extract	0.84 g/kg; 30 days	High-fat diet/STZ-induced diabetic rats	T2D	Yes	Serum	(↑) LDL/VLDL, isoleucine, valine, NAG, acetoacetate, glutamate; (↓) betaine, glucose	[32]
*Astragalus radix* and *Dioscoreae rhizoma*	6.3 g/kg; 4 weeks	High-fat diet/STZ-induced diabetic rats	T2D	Yes	Serum	(↑) taurine, glycine, glutamine; (↓) lipid, pyruvate, TMAO, glycerol, isoleucine, leucine, valine, glucose, tyrosine, 3-hydroxybutyrate, acetoacetate, succinate, xanthine	[33]
Chickpea extract	3 g/kg; 4 weeks	High-fat diet/STZ-induced diabetic rats	T2D	Yes	Cecum	(↑) acetate, propionate, butyrate	[34]
*Acanthopanax sessiliflorus* fruits	3 mg/kg; 4 weeks	High-fat diet-induced mouse model	T2D	Not mentioned	Liver	(↑) formate, inosine, pyroglutamate, taurine; (↓) alanine, tyrosine	[35]
*Enteromorpha prolifera* polysaccharide	450 mg/kg; 12 weeks	High-fat diet-fed hamsters	T2D	Not mentioned	Serum	(↑) arginine; (↓) 2-hydroxyisovalerate, 2-oxoglutarate, 3-hydroxybutyrate, 3-hydroxyisobutyrate, betaine, citrate, glucose, lactate	[36]
*Berberis vernae* extract	0.84 g/kg; 30 days	High-fat diet/STZ-induced diabetic rats	T2D	Yes	Serum	(↑) LDL/VLDL, isoleucine, valine, lipid, NAG, acetoacetate; (↓) TMAO, betaine, glucose	[37]

^a^ Metabolic changes after TCM treatment relative to non-treated diabetes.

## Data Availability

No new data were created or analyzed in this study. Data sharing is not applicable to this article.
